# THINK OF THE SITUATION IN A POSITIVE LIGHT: COGNITIVE REAPPRAISAL, AFFECTIVE REACTIVITY, AND HEALTH

**DOI:** 10.1093/geroni/igac059.480

**Published:** 2022-12-20

**Authors:** Jessica Maras, Kate Leger

**Affiliations:** University of Kentucky, Lexington, Kentucky, United States; The University of Kentucky, Lexington, Kentucky, United States

## Abstract

Cognitive reappraisal is an emotion regulation strategy that benefits health and well-being, but it is currently unclear why this relationship exists. The current study examined how adults react affectively to daily stressful events as a mediating pathway between cognitive reappraisal and health and well-being outcomes across a 20-year period. Participants (N = 1,814) completed waves 1-3 of the Midlife in the United States survey series and the National Study of Daily Experiences, an 8-day daily diary. Results found that negative affective reactivity to daily stressors mediated the relationship between cognitive reappraisal and future health outcomes including the development of chronic conditions, functional limitations, and affective disorders. Exploratory analyses revealed that specific negative emotions, including being nervous and irritable explained this relationship. Findings from this study suggest that strengthening cognitive reappraisal skills may be a good way to reduce affective reactions to daily stressors and enhance long-term health and well-being outcomes.

